# The Amazonian kambô frog *Phyllomedusa bicolor* (Amphibia: Phyllomedusidae): Current knowledge on biology, phylogeography, toxinology, ethnopharmacology and medical aspects

**DOI:** 10.3389/fphar.2022.997318

**Published:** 2022-10-06

**Authors:** Thais A. C. Nogueira, Igor Luis Kaefer, Marco A. Sartim, Manuela B. Pucca, Jacqueline Sachett, André L. Barros, Moysés B. A. Júnior, Djane C. Baía-da-Silva, Paulo S. Bernarde, Hector H. F. Koolen, Wuelton M. Monteiro

**Affiliations:** ^1^ Departamento de Ensino e Pesquisa, Fundação de Medicina Tropical Dr. Heitor Vieira Dourado, Manaus, Amazonas, Brazil; ^2^ Grupo de Pesquisas em Metabolômica e Espectrometria de Massas, Universidade do Estado do Amazonas, Manaus, Amazonas, Brazil; ^3^ Instituto de Ciências Biológicas, Universidade Federal do Amazonas, Manaus, Amazonas, Brazil; ^4^ Departamento de Pós-Graduação, Universidade Nilton Lins, Manaus, Amazonas, Brazil; ^5^ Curso de Medicina, Universidade Federal de Roraima, Boa Vista, Roraima, Brazil; ^6^ Departamento de Ensino e Pesquisa, Fundação Alfredo da Matta, Manaus, Amazonas, Brazil; ^7^ Instituto de Ciências Exatas e Tecnologia, Universidade Federal do Amazonas, Itacoatiara, Amazonas, Brazil; ^8^ Laboratório de Herpetologia, Campus Floresta, Universidade Federal do Acre, Cruzeiro do Sul, Acre, Brazil

**Keywords:** bicoulored tree-frog, giant leaf frog, giant monkey frog, kapum, kampô, two-colored leaf frog, vacina do sapo, frog vaccine

## Abstract

*Phyllomedusa bicolor* (Phyllomedusidae), popularly known as the kambô in Brazil, is a tree frog that is widely distributed in South American countries and is known for producing a skin secretion that is rich in bioactive peptides, which are often used in indigenous rituals. The biological effects of the skin secretion were observed in the first studies with indigenous communities. Over the last six decades, researchers have been studying the chemical composition in detail, as well as the potential pharmacological applications of its constituents. For this reason, indigenous communities and health agents fear the misuse of the kambô, or the inappropriate use of the species, which can result in health complications or even death of users. This article seeks to provide a transdisciplinary review that integrates knowledge regarding the biology of *P. bicolor*, ethnoknowledge about the ritual of the kambô, and the chemistry and pharmacology of the skin secretion of this species, in addition to medical aspects of the indiscriminate use of the kambô. Furthermore, this review seeks to shed light on perspectives on the future of research related to the kambô.

## 1 Natural history of *Phyllomedusa bicolor*

### 1.1 The species: General aspects

*Phyllomedusa bicolor* is popularly known in English as the bicolored treefrog, giant monkey frog, giant leaf frog, two-colored leaf frog, waxy-monkey treefrog, and kambô (and also kampô) in South American countries (Frost, 2021). The word kambô commonly has two meanings in the literature. It can be used for the treefrog *P. bicolor* and also for the shamanic indigenous ritual (Caramaschi and Cruz, 2002; Frost, 2021). Its skin secretion has been used for centuries by the natives of the Western Amazon in shamanic healing and purification rituals known as kambô (Erspamer-Falconieri et al., 1970) or kapum or the “vacina do sapo” (toad vaccine in English). However, this practice has expanded to urban centers as an alternative to conventional medicine (Silva et al., 2019). It is a pharmacologically important taxon because it is considered a rich source of biologically active peptides, many of which have already been identified or characterized, although it is estimated that there are numerous compounds yet to be discovered (Thompson and Williams, 2022).

*Phyllomedusa bicolor* was taxonomically described from Suriname by Boddaert (1772) as *Rana bicolor* and, later, Wagler (1830) proposed the genus *Phyllomedusa* to house this species. Therefore, *P. bicolor* is the type species of its genus. The name *Phyllomedusa* comes from the Greek “phyllo” (leaf or foliage) and “medousa” (queen or protector), therefore meaning “queen of the foliage” or “guardian of the foliage” (Caramaschi and Cruz, 2002). Its specific name “bicolor” refers to the two main colors of this frog (Boddaert, 1772): dorsum dark green and belly white to yellow-white or cream. Monophyly of the genus *Phyllomedusa* is strongly supported by Duellman et al. (2016), and the genus currently contains 16 taxa (Frost, 2021), with *Phyllomedusa chaparroi* being the most recently described (Castroviejo-Fisher et al., 2017). *Phyllomedusa bicolor* and *Phyllomedusa vaillantii* are frequently recovered as sister species that share the presence of osteoderms in the skin dorsum (Ruibal and Shoemaker, 1984; Faivovich et al., 2005; Wiens et al., 2006; Moen and Wiens, 2009; Faivovich et al., 2010; Duellman et al., 2016).

### 1.2. Seasonality, reproductive biology, and life cycle

**FIGURE 1:**
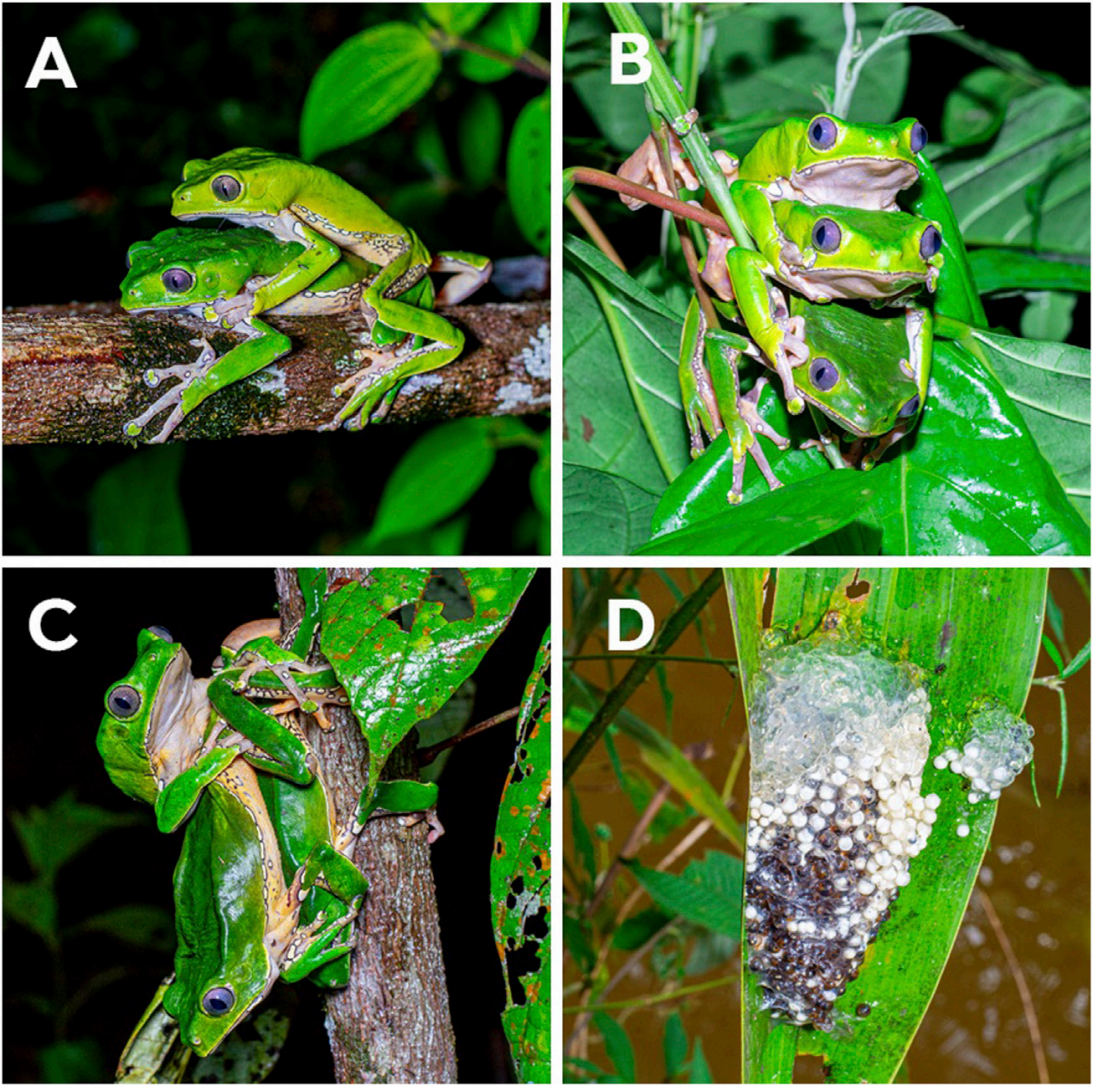
Reproductive behavior of *Phyllomedusa bicolor* in Amapá State, Brazil. **(A)** An axillary amplexus near riparian vegetation. **(B)** Multiple amplexus of two males and one female. **(C)** Physical dispute between two males for a female. **(D)** The spawn on a totally open leaf above a temporary pond of lentic water where tadpoles develop until metamorphosis. Photos: Wirley Almeida.

Reproduction occurs mainly during the rainy season, and the length of reproduction varies geographically throughout the species’ distribution, and also because of increasingly frequent extreme climatic events such as El Niño and La Niña (Lima et al., 2012; Rocha et al., 2021). In the Central Amazon, breeding occurs mostly from November to May (Lima et al., 2012), during which constant and heavy rainfall creates suitable lentic environments such as ponds for development of the tadpoles (Venâncio and Melo-Sampaio, 2010; Pinto et al., 2013). This prolonged reproductive season of the species permits male territoriality, asynchronous arrival of females to breeding sites, as well as male-male competition for females with physical combat that includes body displacement of amplectant pairs (Figure 1C; Souza 2009; Venâncio and Melo-Sampaio, 2010; Silva et al., 2020). Reproduction promotes vocalization and movement through the vegetation, possibly increasing detectability of adult individuals, mostly males, by humans.

Males call alone or in small congregations, on vegetation (branches of bushes and trees), close to streams, temporary or permanent ponds on the inside and edges of the forest, between one and 10 m above the ground (Souza, 2009). The vocalization usually begins at dusk and lasts until near dawn, at which time the males leave the calling sites and move to their upper diurnal shelters (Souza, 2009). The courtship of the species begins at night in upland forests with an active calling male from a high tree until the arrival of the female; after which the pair descends to lower arboreal strata (Venâncio and Melo-Sampaio, 2010; Silva et al., 2020). The advertisement call of *P. bicolor* males is short, loud, sparse, and low-pitched when compared to most treefrogs (Rodriguez and Duellman, 1994; Lima et al., 2012). As the female comes closer, the male climbs on her dorsum to form the axillary or cephalic amplexus (Figure 1A) in which the pair stays for some minutes before the female moves to a suitable oviposition site (Venâncio and Melo-Sampaio, 2010). The oviposition site is chosen on the vegetation above lentic water surfaces (average 70 cm) or near a slow-flowing water stream (Figure 1D) where the mating pair fold leaves (usually tree leaves) to form a chamber to protect the spawn, which is probably a strategy against predation and dehydration (Venâncio and Melo-Sampaio, 2010; Lima et al., 2012; Pinto et al., 2013). However, it is known that the predation rate upon *P. bicolor* spawns can reach up to 61% (Neckel-Silveira and Washlewski, 2004).

**FIGURE 2:**
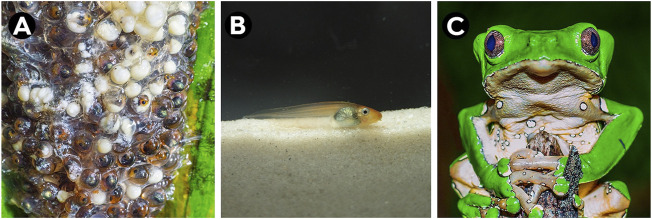
Different ontogenetic stages of *Phyllomedusa bicolor*: **(A)** eggs, **(B)** larvae (tadpole) and **(C)** adult. Photos: A and **(C)** Wirley Almeida; **(B)** Domingos Rodrigues.

The spawn of *P. bicolor* is one of the largest among treefrogs in the Amazon, and corresponds to a gelatinous mass with relatively large (average 2.6 mm) eggs that range between 241 and 1,722 units (Neckel-Oliveira and Wachlevski, 2004; Venâncio and Melo-Sampaio, 2010; Silva et al., 2020) (Figure 2B). After the oviposition, the lecithotrophic eggs develop into tadpoles within the leaf chambers (Pinto et al., 2013). After approximately 2 weeks, tadpoles with external gills emerge from the eggs (Figure 2A) and fall on the water surface under the oviposition site to continue their development with exotrophic feeding until metamorphosis (Figure 2C) (Venâncio and Melo-Sampaio, 2010; Pinto et al., 2013).

### 1.3 Morphology and behavior

*Phyllomedusa bicolor* are relatively large-sized tree frogs: adult males measure 91–118 mm from snout to vent and females 106–119 mm (Souza, 2009; Lima et al., 2012; Duellman et al., 2016). Adults have a dark green dorsum and a white to yellow-white or cream belly (Lima et al., 2012). There are white spots with dark outlines sparsely distributed on the lower lips, chest, and front legs, though more densely distributed on the flanks and hind legs, dark gray iris, a prominent gland that extends from behind the eyes over the tympanum, and vomerine teeth (Caramaschi and Cruz, 2002; Lima et al., 2012). Fingers are transparent brown with large green adhesive discs although little or no webbing on the feet and none on hands (Faivovich et al., 2010; Duellman et al., 2016). The individuals usually exhibit an elegant slow walk over branches and leaves despite being able to jump (Caramaschi and Cruz, 2002). The species of Phyllomedusiae are regarded as photogenic and charismatic animals, and are frequently called “poster frogs” (Faivovich et al., 2010). Probably because of its large size, wide distribution, and its use in shamanic rituals, *P. bicolor* frequently figures as a symbol of the Amazonian fauna (Bernarde, 2012). Phyllomedusiae actively produce skin secretions and frequently spread them throughout the body with the hind and front legs (Blaylock et al., 1976).

Most of the characteristics of the tadpoles are similar to other species of *Phyllomedusa*, i.e., they are exotrophic, lentic, and suspension-raspers (Altig and McDiarmid, 1999; Pinto et al., 2013). The tadpole reaches a total length of up to 50 mm (Souza, 2009). The coloration in life is orange on the dorsum and anterior part of the body, with a silver belly, a pale orange color on the tail musculature and cord, a translucent orange color on the fins, and a silver iris (Figure 2A) (Pinto et al., 2013). This species’ tadpole can be easily distinguished from others (except for those of *Phyllomedusa vaillantii*) due to their unique cord that is present at all stages, and which corroborates the phylogenetic proximity of these two species (Faivovich et al., 2010). *Phyllomedusa bicolor* tadpoles show conspicuous colorations in the dorsal area while ventrally they are considered inconspicuous. This is probably for targeting aerial or terrestrial predators while remaining cryptic for underwater animals (Thibaudeau and Altig, 2012; Pinto and Amézquita, 2022). They also prefer to remain on the surface of the water, displaying their coloration: bigger tadpoles present this behavior whereas smaller individuals tend to be distributed along the water column, thus reinforcing the antipredatory strategy (Pinto and Amézquita, 2022). There is evidence of the toxicity of *P. bicolor* tadpole for vertebrates; it has been shown to significantly affect mice after ingestion, even though Odonata naiads (damselfly larvae) were able to prey on them without difficulties. Therefore, it is likely that the same peptides secreted as adults are present during larval growth (Delfino et al., 1998; Pinto and Amézquita, 2022).

### 1.4 Geographic distribution and genetic variability

**FIGURE 3:**
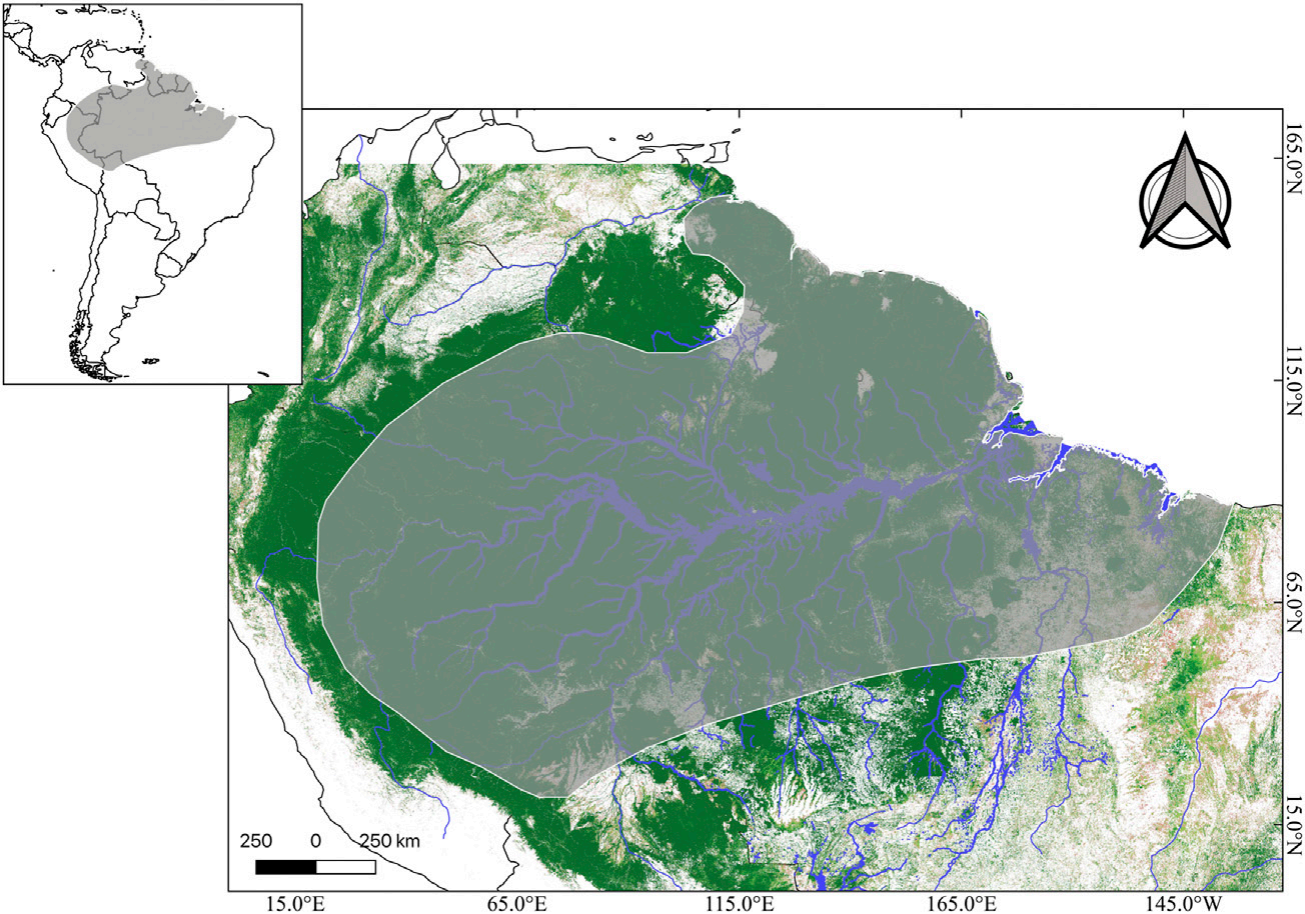
Distributional range of *Phyllomedusa bicolor* (dark gray) in the context of South America (upper panel). Tree coverage is shown in dark green, while lighter green and beige represents less tree coverage. Major Amazonian rivers are depicted in blue. Credits: Shapefile of distributional range from IUCN, and tree cover raster (Landsat 8) from Global Land Analyses & Discovery.

This species has a wide distribution when compared to most amphibian species, since it is found in tropical rainforests in the Amazon Basin in Bolivia, Peru, Colombia, Venezuela, Guyana, French Guiana, Suriname, and possibly Ecuador (Ouboter and Jairam, 2012; Frost, 2021) (Figure 3). However, most of its distributional range lies within Brazil, where the species can be found in rainforests and even in savannah-like environments of all its northern states (Mota et al., 2020; Frost, 2021) (Figure 3).

Despite its large body size, wide distribution and medical importance, many of its few published DNA sequences are not assigned to any specific geographic location, which is due to them being obtained from pet trade specimens (Mota et al., 2020). Surprisingly, even phylogenetic studies on the family lack samples of *P. bicolor* (Wiens et al., 2005; Gomez-Mestre et al., 2008). One study, on the spatial distribution of its genetic variability using both mitochondrial and nuclear markers, detected two well-supported monophyletic groups that diverged approximately 5.16 million years ago in two geographic mega-regions, namely the Eastern and Western Amazon; the former consisting of three highly-structured population groups distributed in the Guiana and Brazilian Shields (Mota et al., 2020). Given that this study was restricted to Brazilian locations and lacked samples from Suriname, the species’ type locality, it is likely that the nominal species is composed of at least two, or possibly more, cryptic species (Mota et al., 2020; Frost, 2021).

### 1.5 Ecological relationships: Diet, predation and parasitism

There appears to be no records of *P. bicolor* prey under natural conditions. Other phyllomedusids show generalist diets and are regarded as opportunistic sit-and-wait predators, consuming mainly arachnids (spiders and mites), coleopterans, and lepidopteran larvae (Bertoluci, 2002; Lima et al., 2012).

The risk of predation of anurans was reported to be smaller in species with chemical defense or large-sized bodies (Shine, 1979), which is the case of the adult *P. bicolor* (Venâncio and Melo-Sampaio, 2010). There are no records of adult *P. bicolor* being preyed on or found among the stomach contents of any taxa that usually preys on treefrogs, such as snakes, birds, bats, or other mammals. This may be due to its large body size and cryptic coloration that helps camouflage it. However, its skin secretion may play a strong role in predation avoidance because it is unpleasant to predators, possibly causing regurgitation, modifying cardiac function, or producing catalepsy (Sazima, 1974; Negri et al., 1992).

Neckel-Oliveira and Wachlevski (2004) observed the spawns of *P. bicolor* being predated by flies, beetles, and mammals, and also reported that spawn predation is common in *Phyllomedusa*. Snakes are known to predate on anuran eggs and may be responsible for some missing clutches (Warkentin, 1995; Martins and Oliveira, 1998; Neckel-Oliveira and Wachlevski, 2004). Neckel-Oliveira and Wachlevski (2004) observed marks of predation of clutches by monkeys. *Phyllomedusa bicolor* tadpoles were recorded among the stomach contents of the aquatic frog *Pipa arrabali,* and this species was also observed actively preying on tadpoles as soon as they dropped from the arboreal nest to the pond (Buchacher, 1993).

Only nine species of nematodes have been reported as parasites of Phyllomedusidae, whereas the genus *Neocosmocercella* was only observed in phyllomedusids, and is likely specific to this family (Campião et al., 2014; Santos et al., 2017). The parasite *Neocosmocercella fisherae* was the only species found in the large intestine of *P. bicolor* specimens (Santos et al., 2017). There was also another report of a filarial parasite located near the lungs of *P. bicolor*, though without further identification (Chai, 2015). Besides the predation of the eggs by flies, there are no records of myiasis for *P. bicolor*. Observation of endoparasites, both intestinal and pulmonary, are more common in *P. bicolor* when compared to ectoparasites, which may reflect the protection conferred by its epidermal secretions against external parasitism.

### 1.6 Conservation and welfare

Amazonian anurans with wide distribution, such as *P. bicolor,* usually present a cryptic taxonomic diversity, which means that species considered not to be under threat may have an inaccurate, and often underestimated conservation status (Gehara et al., 2014; Mota et al., 2020). Despite the wide currently known geographical distribution of *P. bicolor*, the species is not abundant in faunal inventories and ecological studies, and is regarded as a less abundant taxon in anuran assemblages (Menin et al., 2008; Jairam, 2019). In spite of this, *P. bicolor* is not classed as threatened in the Convention on International Trade in Endangered Species of Wild Fauna and Flora (CITES) appendix; an international arrangement that aims to ensure that international trade of wild animals and plants does not threaten the survival of species (https://cites.org/eng). According to the International Union for Conservation of Nature’s (IUCN) Red List of Threatened Species, which indicates the species’ global conservation status (and urgently needs updating), *P. bicolor* is listed as a species of least concern because of its large distributional range and occurrence in protected areas (IUCN, 2021).

The aforementioned conservation status of *P. bicolor* might be underestimated due to the following causes: 1) Specific habitat requirements, since it is highly intolerant of anthropized habitats, and is usually found in extensive and well-preserved forests (Tsuji-Nishikido and Menin, 2011; Lima et al., 2012). Their reproductive behavior is elaborate, requiring suitable terrestrial and aquatic (especially lentic) environments for the completion of its reproductive cycle; 2) Possible cryptic diversity with species occurring outside protected areas and/or with small distributional ranges (Mota et al., 2020); 3) Unknown populational effects due to manipulation of individuals for venom extraction, which sometimes involves the use of electric shocks (Long et al., 2017; Liu et al., 2020; Chen et al., 2021), and restraints (Den Brave et al., 2014). In addition, it is unknown how the manipulation and/or removal of adult individuals from nature by humans affects individual behavior, including parental care and, consequently, offspring survival in *P. bicolor*; 4) Underreporting of trafficked individuals in the pet trade. As any other illegal substance, there is a high risk of identification error, especially due to the morphological similarity among *Phyllomedusa* species, or possible adulteration of the lyophilized skin extracts (Lima and Labate, 2007; Silva et al., 2019). For example, the metropolitan region of Manaus is known to contain at least four species of *Phyllomedusa* occurring in sympatry (same location) and syntopy (similar microhabitat) (Lima et al., 2012), which may lead to the misidentification of species and extraction of skin secretions of non-targeted species.

*Phyllomedusa bicolor* is mostly distributed in Brazil, a country with restrictive legislation against the use and advertisement of kambô for medical purposes without proper clarification of its risks and the lack of evidence for medical treatments (ANVISA, 2004). In addition, there are strong restrictions in regards access to wild animals and their products, even by scientific researchers (IBAMA/ICMBIO). Despite this, the main centers where the secretion is applied are located outside the distribution area of the species (Iakp, 2022; Thompson and Williams, 2022), and there is no information on how individuals and secretions reach these sites since the exact origin and taxonomic accuracy of the sampled individuals is rarely mentioned at the application centers. In 2013, the Regional Superintendency of the Federal Police of the Brazilian State of Acre developed a technique to chemically identify the “vacina do sapo” (toad vaccine) and inhibit trafficking and smuggling, but there is no information regarding the current application of such a technique.

## 2 The poisonous secretion of *Phyllomedusa bicolor*

### 2.1 Skin structure of *Phyllomedusa bicolor*

**FIGURE 4:**
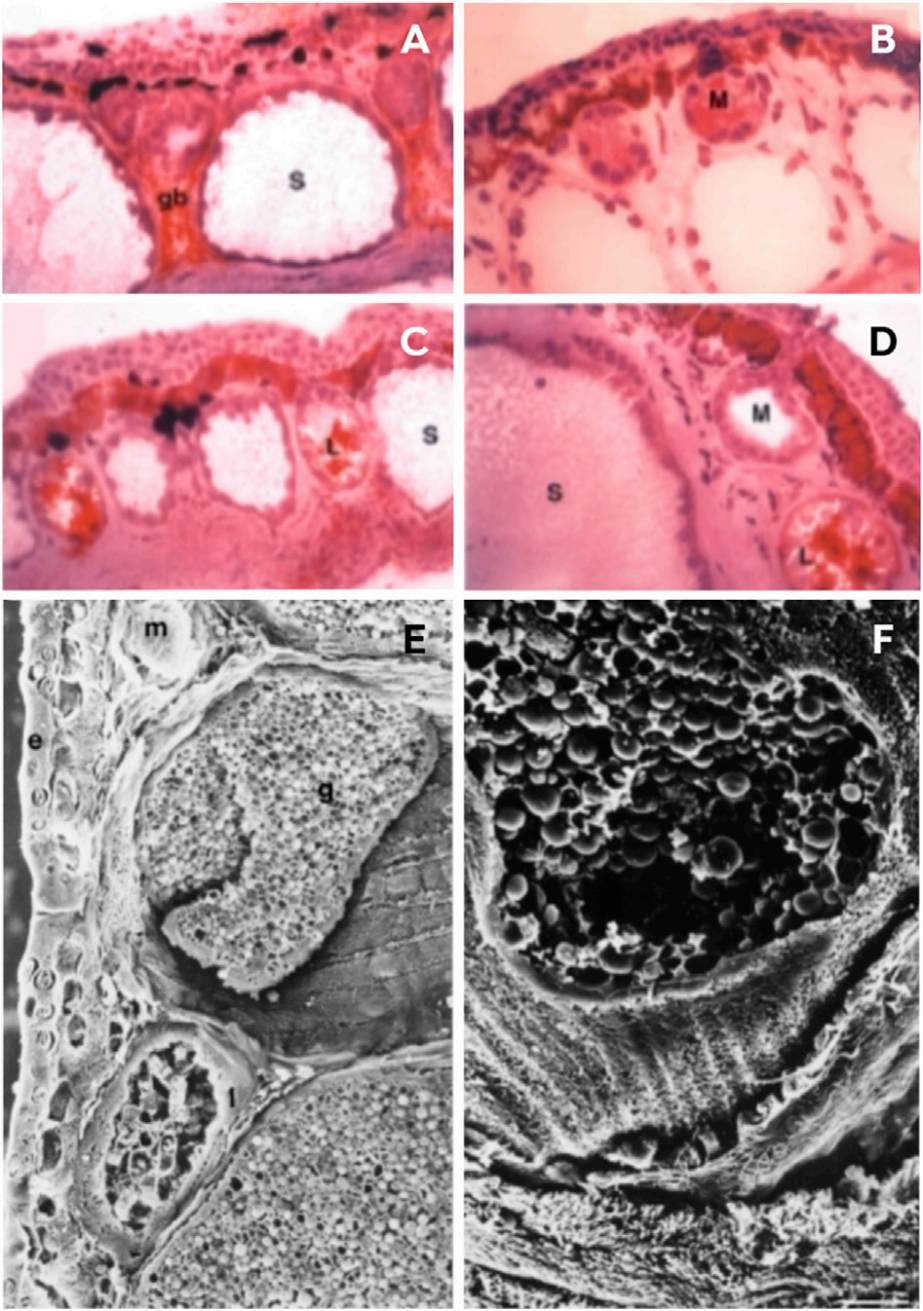
Skin structure of Phyllomedusa bicolor. **(A–D)** Sections of paraffin-embedded material at different stages of development; **(E–F)** Cryostat sections immunolabeled with I-13 antibodies by phase contrast. **(A)** A tadpole without a developed lipid gland, but showing a rudimentary lipid gland between two serous glands. **(B–C)** A juvenile immediately after metamorphosis; the serous glands are not so voluminous and not so deep in the dermis. **(D)** Adult stage with mature serous glands. **(E–F)** Sections of adult skin structure *via* scanning electron micrography. **(E)** A dorsolateral view indicating the glands in the dermis: **(E)** epidermis, **(G)** serous gland, **(L)** lipid gland, (m) mucous gland. **(F)** Serous glands broken and releasing spherical secretion granules. Photos: Adapted from Lacombe et al. (2000) (License number 5337320779774, Elsevier, Amsterdam, Netherlands).

Amphibian skin is a thin, permeable and flexible integument that is highly vascular and is responsible for water absorption, respiration, thermoregulation, osmoregulation and physical and chemical defense against predators and desiccation (Wager, 1986; Clarke, 1997; Larsen and Ramløv, 2013). It consists of the layers of the *epidermis* and dermis, and there are countless glands present on the entire epidermal surface of frogs (Figure 4) that constantly produce and secrete small amounts of poison (Jared et al., 2009; Govender et al., 2012). Among amphibians, arboreal frogs, such as treefrogs, spend most of their life in humid environments, where there is greater exposure of individuals to infectious diseases (Liu et al., 2021). The anuran’s skin adapts to different environmental pressures of the species’ habitat, thus resulting in great morphofunctional diversity (Toledo and Jared, 1993; Barra and Simmaco, 1995). Their secretions play an essential role in aiding cutaneous respiration, reproduction, defense against predators, as well as defense against desiccation and proliferation of microorganisms (Toledo and Jared, 1995).

Lacombe et al. (2000) conducted an ultrastructural study that characterized the skin of the species of *Phyllomedusa* according to size and activity, and presented a profile of skin glands composed of three types of cutaneous glands: lipid, mucous, and serous (Figure 4). These glands are located deep in the skin and subcutaneous connective tissue, although the serous glands are larger and lie deeper than the others (Lacombe et al., 2000). Lipid glands are mostly located in the dorsal and dorsolateral regions and are responsible for impermeabilization of the skin, and prevent water loss through desiccation (Lacombe et al., 2000; Castanho and De Luca, 2001). Mucous glands are mainly distributed in the ventral region and are practically absent from the back. These produce mucus to assist the cutaneous physiological functions such as respiration, reproduction, defense and thermoregulation (Toledo and Jared, 1995; Lacombe et al., 2000). Serous glands are the largest and are distributed all over the body; however, they are prominent in the dorsolateral region, and behind the eyes, and form the parotid glands (Lacombe et al., 2000). These glands are primarily responsible for passive defense and are the first to develop, although the gland duct lengthens according to the maturation of the *epidermis*, and opens to the skin surface once they reach metamorphosis (Toledo and Jared, 1995; Lacombe et al., 2000). According to Lacombe et al. (2000), there are two classes of serous glands, type I and II. The first type has a poorly developed smooth endoplasmic reticulum, which is divided into two subtypes, Ia and Ib. Type Ia is characterized by dense granules that carry out the biosynthesis of proteinaceous products reserved for exocytosis (Figure 4H), and involves both the rough endoplasmic reticulum and the Golgi apparatus (Delfino, 1991). The serous secretion of type Ib shows vesicles carrying a lucent material that undergoes maturation without condensation (Toledo and Jared, 1995). Type II is typical of *P. bicolor* since they present a well-developed, smooth, endoplasmic reticulum that probably plays a role in the biosynthesis of peptides (Blaylock et al., 1976; Lacombe et al., 2000). These peptides are first synthesized as prepropeptides and then, to mature these peptides, the removal of the peptide signal and the acidic propiece occurs and, later, the mature peptides are stored in round granules (Lacombe et al., 2000; Nicolas and El Amri, 2009; Calderon et al., 2011).

### 2.2 Composition of *Phyllomedusa bicolor* skin secretion

Amphibians inhabit microorganism-rich environments, especially treefrogs that live in a very humid environment for the main part of their life. As a result, they are more susceptible to pathogens and, in order to defend themselves, they produce potent antimicrobial peptides (Daly, 1995; Govender et al., 2012; Liu et al., 2021). The innate immunity system has the main responsibility for individual survival, in which the secretion produced by anuran glands inhibits diseases, and is toxic to other animals, which may prevent predation in some cases (Nicolas and Mor, 1995; Kimbrell and Beutler, 2001; Toledo et al., 2011). The release of the skin secretion is considered a very efficient defense mechanism because it provides unpalatable characteristics and/or toxicity against potential predators (Bevins and Zasloff, 1990; Daly, 1995; Williams et al., 2000; Rodriguez et al., 2011; Saporito et al., 2012; Hantak et al., 2013). Among the chemical substances responsible for anuran defense, peptides play a central role, while alkaloids are less common and are released in the tegument by some species (Saporito et al., 2009; Jeckel et al., 2015). Peptides are mostly known to act against pathogenic microorganisms; however, studies have shown that they can also have functions related to the interaction between species, such as bradykinin-like peptides released by *Bombina orientalis* (Bombinatoridae), which are known to be facilitators in intoxication process of predators (Rödel et al., 2013; Raaymakers et al., 2018). Even though the skin secretions of *Phyllomedusa bicolor* are widely known as a rich source of biologically-active peptides, so far, studies have not been carried to disccover whether the substances released in the skin secretions possess toxicity against predators (Bevins and Zasloff, 1990; Daly, 1995).

All species of *Phyllomedusa* are prolific producers of peptides, of which, the most representative family is the dermaseptins (Erspamer et al., 1985). Since the first record of the isolation of a peptide from this genus (dating from 1966), more than 277 peptides with unique sequences have been described (Calderon et al., 2011). Most of the bioactive peptides so far characterized have displayed potential applications in medicine, such as phyllocaeruleins with hypotensive properties, tachykinins and phyllokinins as vasodilators, dermorphins and deltorphins with opiate-like properties, and adenoregulins with antibiotic properties (Daly et al., 1992; Mor et al., 1994a; Lacombe et al., 2000; Hesselink and Sacerdote, 2019). These peptides are placed in groups according to their primary activity: antimicrobial peptides; smooth muscle active peptides; and nervous system active peptides (Erspamer et al., 1981). Due to the numerous biological activities of these substances and the similarities with the amino acid sequences related to mammalian neuropeptides and hormones, many have aroused the interest from a medical and pharmacological perspective, such as in the production of new drugs (Lazarus et al., 1999; Basir et al., 2000; Chen et al., 2002; Doyle et al., 2002; Severini et al., 2002; Conceição et al., 2006).

The venom of *P. bicolor* is used by natives in traditional shamanic rituals in the southwestern Amazon, and is reported to be used as a stimulant and also for healing purposes, although there is no conclusive scientific evidence of its effectiveness (Erspamer-Falconieri et al., 1970; Majic et al., 2021). So far, from the skin secretions of *P. bicolor*, only peptides have been characterized, from which 28 different sequences have been determined, with most of them belonging to the dermaseptin, deltorphin and demorphin families (Thompson and Williams, 2022).

#### 2.2.1 Dermaseptins

Dermaseptins constitute a superfamily of antimicrobial cationic peptides that are produced in the skin of Phyllomedusidae. They are known to be genetically related, and similar in precursor signals and sequences, but have since diversified and given rise to structurally different peptide subfamilies (Nicolas and El Amri, 2009). Dermaseptins are derived from precursors that have a highly conserved N-terminal preprosequence (Nicolas and Amiche, 2006). Dermaseptins have related structures that differ in sequence length, as well as differences in amino acid sequences. The first dermaseptin characterized was S1 (DRS1), which is present in the skin of *P. sauvagii* (Mor et al., 1991) while, for *P. bicolor,* the dermaseptins isolated/characterized in its tegument, so far, belong to the B series (B and B1-B6), G (G3) and other classes of dermaseptin-like peptides (Table 1). With the exception of DRS-S4, all dermaseptins have lower or no toxicity to mammalian cells (Ladram and Nicolas, 2016). The selectiveness and the ability to destabilize the plasmatic membrane is the most pertinent characteristic of the antimicrobial action of dermaseptins. This family of antimicrobial peptides is mainly responsible for the anti-infective passive defense barrier, in which the killing mechanism acts fast by destroying pathogen’s plasmatic membrane in a few minutes, and is unlikely to cause antibiotic-resistance (Shai, 1995; Feder et al., 2000).

**TABLE 1:** Classification, sequence details and pharmacological activities and effects of peptides from *Phyllomedusa bicolor*.

Name	Sequence/Class^a^	Pharmacological properties and effects^b^ ^,^ ^c^
	Dermaseptins *stricto sensu*	
Dermaseptin B	Asp-Val-Leu-Lys-Lys-Ile-Gly-Thr-Val-Ala-Leu-His-Ala-Gly-Lys-Ala-Ala-Leu-Gly-Ala-Val-Ala-Asp-Thr-Ile-Ser-Gln-NH_2_	Antimicrobial^c^
Dermaseptin B1	Ala-Met-Trp-Lys-Asp-Val-Leu-Lys-Lys-Ile-Gly-Thr-Val-Ala-Leu-His-Ala-Gly-Lys-Ala-Ala-Leu-Gly-Ala-Val-Ala-Asp-Thr-Ile-Ser-Gln-NH_2_	Antimicrobial^c^
Dermaseptin B2	Gly-Leu-Trp-Ser-Lys-Ile-Lys-Glu-Val-Gly-Lys-Glu-Ala-Ala-Lys-Ala-Ala-Ala-Lys-Ala-Ala-Gly-Lys-Ala-Ala-Leu-Gly-Ala-Val-Ser-Glu-Ala-Val-NH_2_	Antimicrobial^c^, antitumor^c^
Dermaseptin B3	Ala-Leu-Trp-Lys-Asn-Met-Leu-Lys-Gly-Ile-Gly-Lys-Leu-Ala-Gly-Gln-Ala-Ala-Leu-Gly-Ala-Val-Lys-Thr-Leu-Val-Gly-Ala-OH	Antimicrobial^c^, antitumor^c^
Dermaseptin B4	Ala-Leu-Trp-Lys-Asp-Ile-Leu-Lys-Asn-Val-Gly-Lys-Ala-Ala-Gly-Lys-Ala-Val-Leu-Asn-Thr-Val-Thr-Asp-Met-Val-Asn-Gln-NH_2_	Antimicrobial^c^, antidiabetic^c^
Dermaseptin B5	Gly-Leu-Trp-Asn-Lys-Ile-Lys-Glu-Ala-Ala-Lys-Ser-Ala-Gly-Lys-Ala-Ala-Leu-Gly-Phe-Val-Asn-Glu-Met-Val-NH_2_	
Dermaseptin B6	Ala-Leu-Trp-Lys-Asp-Ile-Leu-Lys-Asn-Ala-Gly-Lys-Ala-Ala-Leu-Asn-Glu-Ile-Asn-Gln-Leu-Val-Asn-Gln-NH_2_	Antimicrobial^c^
Dermaseptin G3	Ala-Leu-Trp-Lys-Thr-Ile-Ile-Lys-Gly-Ala-Gly-Lys-Met-Ile-Gly-Ser-Leu-Ala-Lys-Asn-Leu-Leu-Gly-Ser-Gln-Ala-Gln-Pro-Glu-Ser-OH	Antimicrobial^c^
	*Other dermaseptins*	
Adenoregulin	Gly-Met-Trp-Ser-Lys-Ile-Lys-Glu-Ala-Gly-Lys-Ala-Ala-Ala-Lys-Ala-Ala-Ala-Lys-Ala-Ala-Gly-Lys-Ala-Ala-Leu-Asp-Val-Val-Ser-Gly-Ala-Ile-OH	Antimicrobial^c^, antitumor^c^, agonist of A_1_-adenosine receptors^c^
Dermatoxin	Ser-Leu-Gly-Ser-Phe-Leu-Lys-Gly-Val-Gly-Thr-Thr-Leu-Ala-Ser-Val-Gly-Lys-Val-Val-Ser-Asp-Gln-Phe-Gly-Lys-Leu-Leu-Gln-Ala-Gly-Gln-OH	Antimicrobial^c^
Phylloxin	Gly-Trp-Met-Ser-Lys-Ile-Ala-Ser-Gly-Ile-Gly-Thr-Phe-Leu-Ser-Gly-Ile-Gln-Gln-OH	Antimicrobial^c^
Phylloseptin-B2	Phe-Leu-Ser-Leu-Ile-Pro-His-Ile-Val-Ser-Gly-Val-Ala-Ser-Ile-Ala-Lys-His-Phe-Gly-OH	Antimicrobial^c^
	*Deltorphins*	
[D-Ala^2^]-deltorphin I	Tyr-D-Ala-Phe-Glu-Val-Val-Gly-NH_2_	Catatonia^b^, nausea^b^, vomiting^b^, euphoria^b^ and analgesic^c^
[D-Ala^2^]-deltorphin II	Tyr-D-Ala-Phe-Asp-Val-Val-Gly-NH_2_	Catatonia^b^, nausea^b^, vomiting^b^, euphoria^b^ and analgesic^c^
	*Dermophins*	
[Lys^7^]-dermorphin	Tyr-Asp-Ala-Phe-Gly-Tyr-Pro-Lys-OH	Catatonia^b^, nausea^b^, vomiting^b^, euphoria^b^ and analgesic^c^
[Trp^4^,Asn^7^]-dermorphin	Tyr-Asp-Ala-Phe-Gly-Tyr-Pro-Asn-OH	Catatonia^b^, nausea^b^, vomiting^b^, euphoria^b^ and analgesic^c^
New dermorphin I	Tyr-D-Ala-Phe-Gly-Tyr-Pro-Lys-OH	
New dermorphin II	Tyr-D-Ala-Phe-Trp-Asn-OH	Analgesic^c^
New dermorphin III	Tyr-D-Ala-Phe-Trp-Try-Pro-Asn-OH	
	*Caerulein-like peptides*	
Phyllocaerulein	Pyr-Glu-Tyr(SO_3_H)-Thr-Gly-Trp-Met-Asp-Phe-NH_2_	Lowered blood pressure^b^, nausea and vomiting^b^
	*Corticotropin-releasing hormone-like peptides*	
Sauvagine	Pyr-Gly-Pro-Pro-Ile-Ser-Ile-Asp-Leu-Ser-Leu-Glu-Leu-Leu-Arg-Lys-Met-Ile-Glu-Ile-Glu-Lys-Gln-Glu-Lys-Glu-Lys-Gln-Gln-Ala-Ala-Asn-Asn-Arg-Leu-Leu-Leu-Asp-Thr-Ile-NH_2_	Lowered blood pressure^b^, nausea^b^, vomiting^b^, increased bile secretion^b^, hallucinogen^b^ and Anti-hyperprolactinemia activity^c^
	*Calcitonins*	
Skin calcitonin gene-related peptide (SCGRP)	Ser-Cys-Asp-Thr-Ser-Thr-Cys-Ala-Thr-Gln-Arg-Leu-Ala-Asp-Phe-Leu-Ser-Arg-Ser-Gly-Gly-Ile-Gly-Ser-Pro-Asp-Phe-Val-Pro-Thr-Asp-Val-Ser-Ala-Asn-Ser-Phe-NH_2_	Regulation of calcium levels potential^c^
	*Pancreatic polypeptides*	
Skin peptide tyrosine (SPYY)	Tyr-Pro-Pro-Lys-Pro-Glu-Ser-Pro-Gly-Glu-Asp-Ala-Ser-Pro-Glu-Glu-Met-Asn-Lys-Tyr-Leu-Thr-Ala-Leu-Arg-His-Tyr-Ile-Ans-Leu-Val-Thr-Arg-Gln-Arg-Tyr-NH_2_	Antimicrobial^c^
	*Tachykinins*	
Phyllomedusin	Pyr-Ans-Pro-Ans-Arg-Phe-Ile-Gly-Leu-Met-NH_2_	Skin burns^b^ and inflammation^b^
	*Bradykinins*	
Phyllokinin	Arg-Pro-Pro-Gly-Phe-Ser-Pro-Phe-Arg-Ile-Tyr(SO_3_H)	Skin burns^b^
	*Bombesins*	
Phyllolitorin	Pyr-Leu-Trp-Ala-Val-Gly-Ser-Phe-Met-NH_2_	

a Chemical groups in bold are post-translational modifications.

b Pharmacological efect related to the kambô ritual.

c Other pharmacological property.

##### 2.2.1.1 Dermaseptins *stricto sensu*

As a chemical characteristic, most dermaseptins *stricto sensu* have the C-terminal residue amidated and have the amino acid leucine at position 2 of their primary structure, except dermaseptins B and B1, which have valine and methionine in the same position, respectively. Furthermore, they have a tryptophan residue at position 3 of the primary structure (Daly et al., 1992; Mor et al., 1994a; Charpentier et al., 1998; Fleury et al., 1998; König et al., 2012) (Table 1). Most of the peptides from *P. bicolor* display post-translationally modified amino acid residues, which play an important role in the activity and receptor specificity (Conibear, 2020). In addition, some modifications can enable cross-coupling of specific amino acids, thus yielding derivatives with increased biostability. Furthermore, the inclusion of such modifications can provide resistance against proteolytic degradation, especially for antimicrobial peptides (Tiwari, 2019). As they are α-helical, cationic, and amphipathic, they act directly on the cell membrane of microorganisms, destabilizing the phospholipid bases and preventing the entry and exit of substances, which leads to cell lysis (Nicolas and Amiche, 2006). For *P. bicolor*, seven peptide sequences of the dermaseptin *stricto sensu* type are known (all from the B series), namely dermaseptin B (DRS-B), dermaseptin B1 (DRS-B1), dermaseptin B2 (DRS-B2), dermaseptin B3 (DRS-B3), dermaseptin B4 (DRS-B4), dermaseptin B5 (DRS-B5) and dermaseptin B6 (DRS-B6). The number of B-series dermaseptin *stricto sensu* residues found in *P. bicolor* ranges from 24 to 33 amino acids (Charpentier et al., 1998; Fleury, 1998; König et al., 2012) (Table 1).

The DRS-B peptide was first isolated by Mor et al. (1994a), and presented itself as a peptide amide with 27 residues in its sequence, in which the lysine residues alternate hydrophobic and hydrophilic portions. DRS-B shows 78% similarity with dermaseptin S, differing only by a series of deletions and substitutions (Table 1). Mor et al. (1994a) were the first to test DRS-B against pathogenic fungi and bacteria. Minimum inhibitory concentration (MIC) against eight species of fungi and five species of bacteria were inferred. The antimicrobial potential of DRS-B was evident against the fungi *Cryptococcus neoformans*, *Candida albicans*, *Microsporum canis*, *Tricophyton rubrum*, *T. mentagrophytes*, *Arthroderma simii*, *Aspergillus fumigatus* and *A. niger* and the bacteria *Aeromonas caviae*, *Escherichia coli*, *Enterococcus faecalis* and *Nocardia brasiliensis* since MIC values of between 40 and 60 µM were observed (Mor et al., 1994a).

Likewise, all other dermaseptins *stricto sensu* (DRS-B1 to DRS-B6) have shown activities against microorganisms. DRSB1 has 31 amino acid residues in its primary structure and, unlike other dermaseptins *stricto sensu*, it has a methionine residue at position 2 of the primary sequence. On the other hand, DRS-B2 has 33 amino acid residues in its structure. Differently from what was reported for the other dermaseptins, Van Zoggel et al. (2012) demonstrated that DRS-B2 inhibited the growth of prostatic adenocarcinomas and some other cell lines. DRS-B3, on the other hand, has 28 amino acid residues in its primary structure, but differs from the others as it does not have any modifications at the C-terminal residue. Similar to DRS-B3, the DRS-B4 peptide has the same amount of amino acid residues; however, it does have a modified C-terminal group (Van Zoggel et al., 2012) (Table 1). Both DRS-B3 and DRS-B4 showed antibacterial activities with MICs ranging between 1.3 and 11.6 µM (Van Zoggel et al., 2012) (Table 1). Morevoer, DRS-B4 showed antidiabetic activity, in which the treatment of glucose-responsive BRIN-BD11 cells with this peptide led to the stimulation of insulin release (Marenah et al., 2004).

The DRS-B5 peptide, composed of 25 amino acid residues, has not been evaluated for its biological potential so far, but its antimicrobial potential is suggested, as is the case for other dermaseptins (Charpentier et al., 1998). Likewise, DRS-B6, whose primary sequence has 24 amino acid residues, has also not been studied for its pharmacological potential. DRS-B6, together with DRS-B1, were related to the chemical defense mechanisms of *P. bicolor* since they are secreted by the serous glands located in the tibia (König et al., 2012). Finally, the synthetic dermaseptin, named DRS-G3, was identified through the cDNA technique in *P. bicolor* and synthesized by using 9-fluorenylmethoxycarbonyl-protected (FMOC) amino acids (Fleury et al., 1998). This peptide has 30 residues in its primary structure with a conserved C-terminal. It is possible to note that it has the three amino acid residues (Ala, Leu and Trp) that are common to most of the other dermaseptins present in *P. bicolor*. Fleury et al. (1998) analyzed the biological activity of DRS-G3 and observed that, as with the known dermaseptins, it also has antimicrobial activity against bacteria. The authors tested, in addition to the MIC, the minimum lethal concentration (MLC) necessary to kill bacteria such as Mollicutes, Firmicutes, and Gracilicutes, and found MICs ranging from 3.0 to 6.25 µM and MLCs ranging from 6.25 to 100 µM (Table 1).

##### 2.2.1.2 Other dermaseptins

Adenoregulin is a polycationic peptide composed of 33 amino acid residues in its primary structure (Table 1). This substance has an α-helical structure with lysines arranged on the external face (Daly et al., 1992). When compared to dermaseptins, adenoregulin has a series of substitutions in its amino acid residues, and no modification in the C-terminal. Cao et al. (2005) produced a recombinant adenoregulin derivative by means of heterologous expression in *E. coli*. This derivative differs from the original molecule due to the presence of an additional amidated glutamine residue in the C-terminal, which increased the potency of its antimicrobial activity against *Tritirachium album* and *S. cerevisiae*.

Besides its antimicrobial properties, adenoregulin has antitumor and angiostatic properties at low concentrations. Interestingly, the anticancer activity of this molecule is linked to cell necrosis (Santos et al., 2017). Additionally, the potential in the regulation of cellular metabolism is also mentioned, since this peptide can bind to adenosine A1 receptors, thus making it a potential molecule for the treatment of depression, Alzheimer’s disease, and Parkinson’s disease. (Cao et al., 2005).

Dermotoxin is a peptide derived from the same precursors of the dermaseptin family, and is a cationic and amphipathic peptide with 32 amino acid residues in its primary structure and has no modifications in the C-terminal (Amiche et al., 2000) (Table 1). In *P. bicolor*, it has been observed that dermotoxin is expressed both in the skin, intestines and in the brain. Dermatoxin has effective broad-spectrum antimicrobial activity, as do others from the dermaseptin family. Amiche et al. (2000) tested dermatoxin and observed antibacterial activity with MICs of between 6.25 and 100 µM for the Gram-positive bacteria *Bacillus megaterium* and *Corynebacterium glutamicum*; for bacteria without cell walls *A. laidlawii* and *Spiroplasma melliferum*; and for the Gram-negative bacterium *Sinorhizobium meliloti*.

Phylloxin is also derived from the dermaseptin precursor group, and has 19 amino acid residues in its primary structure without post-translational modifications (Pierre et al., 2000) (Table 1). Pierre et al. (2000) found antimicrobial activity against some strains of bacteria; however, with less inhibition capability than other dermaseptins. Phylloxin was promising against *A. laidlawii*, *Bacillus megaterium*, *Spiroplasma melliferum*, *Escherichia coli*, and *Micrococcus luteus*. Regarding other pharmacological properties, there is no information beyond that related to its antimicrobial activities.

The phylloseptin-B2 peptide was first isolated in Phyllomedusidae from the cutaneous secretion of *P. sauvagii* (Zhang et al., 2010) and, later, an analog was characterized in *P. bicolor* through cDNA cloning (König et al., 2012). As the peptide precursors were not previously identified in *P. bicolor* and the orthology with the related sequences of *P. sauvagii* and *P. hypochondrialis* are not guaranteed, the peptide was designated as phylloseptin-B2, though further studies on the subject should be conducted. These antibiotical properties of plasmatic membrane lysis of the peptides envolve several bubble-like formations that disrupt the membrane (Leite et al., 2005). The antimicrobial activity spectrum comprises Gram-negative bacteria, e.g., *Acinetobacter calcoaceticus*, *E. coli* and *Pseudomonas aeruginosa* (Leite et al., 2005; Resende et al., 2008); Gram-positive bacteria, e.g., *Enterococcus faecalis*, *Klebsiella peneumoniae*, *Staphylococcus aureus* and *Streptococcus agalactiae*; fungi: *C. albicans* (Resende et al., 2008); and protozoa, e.g., *Leishmania amazonensis* (promastigotes) (Kückelhaus et al., 2009), *Plasmodium falciparum* (rings, trophozoites and schizonts) (Kückelhaus et al., 2009), and *Trypanosoma cruzi* (trypomastigotes) (Leite et al., 2005). However, a toxic effect in mammalls cells was observed, but only in extremally high concetrations (Kückelhaus et al., 2007; Kückelhaus et al., 2009) and the effects on blood cells were insignificant (Leite et al., 2005).

#### 2.2.2 Pancreatic polypeptides (PP)

Pancreatic polypeptides (PP) have been isolated during insulin preparation, and are the first of the PP family. Another peptide of this family is the tyrosine peptide (PYY), whose characteristic is the C-terminal amidated tyrosine residue (Grandt et al., 1994). In anurans, PP derivatives are known, especially in the species *Xenopus laevis*, whose function in the organism is still not well understood (Kim et al., 2001).

In the species *P. bicolor*, the only PP characterized so far is the skin peptide tyrosine (SPYY), which was isolated by Mor et al. (1994a). This substance has a primary structure composed of 36 amino acid residues, in which the C-terminal residue is amidated (Table 1). The function of this peptide in *P. bicolor* is still uncertain, but the inhibitory activity against the alpha-melanocyte stimulating hormone (α-MSH) was tested and it was possible to demonstrate that *in vitro* SPYY inhibits the secretion of α-MSH (Mor et al., 1994b). The antimicrobial activity of these peptides comprises strains of Gram-negative bacteria (e.g., *Aerornonas caviae* and *E. coli*), Gram-positive bacteria (e.g., *Enterococcus faecalis* and *Nocardia brasiliensis*), fungi (e.g., *Arthroderma simii*, *Aspergillus fumigatus*, *A. niger*, *Microsporum canis*) and protozoa (e.g., *Leishmania major promastigotes*) (Calderon et al., 2011).

#### 2.2.3 Calcitonins

This family consists of peptides with from 32 to 53 amino acid residues (Hay et al., 2017), which are recognized as hormones that are capable of rapidly reducing circulating calcium levels in the body (Naot et al., 2018). The reduction occurs by inhibiting the efflux of calcium from the bone, since peptides of this class act as potent inhibitors of bone resorption (Naot et al., 2018). Other substances are well known in this family, such as amylin, and adrenoregulin (Hay et al., 2017).

Skin calcitonin gene-related peptide (SCGRP) was isolated from the skin of *P. bicolor* individuals by Seon et al. (2000). The primary structure of this peptide has 37 amino acid residues, and the C-terminal residue is amidated, in addition to having a disulfide bridge between the cysteines at positions two and seven (Table 1). The presence of the disulfide bond may be related to the stabilization of the whole three-dimensional arrangement of SCGRP, which can be crucial for hormonal function (Góngora-Benítez et al., 2014). The researchers who discovered this peptide performed binding assays with the calcitonin receptor and observed that SCGRP differs from all other members of calcitonins in at least nine positions. Like other peptides of this family, it showed competitive inhibition and high specific affinity binding to calcitonin receptors in the brain of rats (Seon et al., 2000).

#### 2.2.4 Corticotropin-releasing hormone-like peptide

Sauvagine is the only peptide in the family of the corticotropin-releasing hormone-like peptides identified in *P. bicolor* (Erspamer et al., 1993). It has 40 amino acid residues in which the C-terminal residue is amidated, as well as the presence of pyroglutamic acid as the N-terminal residue (Table 1). This substance is also related to some effects of kambô, as well as having hypotensive and antidiuretic activities in rats (Erspamer et al., 1981). Promising results were obtained when sauvagine was evaluated against the condition hyperprolactinemia, in which 500 µg of sauvagine, administered by subcutaneous injection, produced an immediate fall of elevated serum prolactin values to normal values within 5 h (Erspamer et al., 1981).

This peptide mimics the functional consequences of stress exposure and acts as a corticotropin-releasing factor (CRF) (Lovejoy and Balment, 1999; Hesselink and Sacerdote, 2019). It has two subtypes of CRF receptor (CRFR) that binds CRFR1 and CRFR2, and it mediates anxiety due to its CRFR2 agonism (Eckart et al., 1999; Hesselink and Sacerdote, 2019). The effects in animal model studies relate a sequence of an intense, long-lasting hypotensive action followed by tachycardia, antidiuresis, decreasing glomerular filtration rate (GFR) and an increase in tubular Na^+^ reabsorption (Montecucchi et al., 1979; Erspamer et al., 1981; Heinrichs and Taché, 2001). Moreover, there is a decrease in body temperature caused by the D2 dopamine receptor-mediated mechanism (Hesselink and Sacerdote, 2019). In dog models, it showed different effects such as long-term increases in blood flow (Lovejoy and Balment, 1999).

#### 2.2.5 Tachykinins

Tachykinins are a family of peptides present in amphibians and mammals that exhibit neurotransmitter activity (Steinhoff et al., 2014). They are expressed throughout the nervous and immune system, and regulate an extraordinarily diverse range of physiological processes, as well as being implicated in important pathological conditions (Steinhoff et al., 2014). In anurans, the presence of tachykinins was first described by Schäffer and Gábriel (2005), who detected the presence of this peptide in the retina of an individual of the genus *Pelobates*. In *P. bicolor,* the only known tachykinin so far is phyllomedusin.

Phyllomedusin is a decapeptide that was first characterized by Anastasi and Esparmer (1970). Interestingly, it has the pyroglutamic amino acid as an N-terminal residue, in addition to being amidated in the C-terminal (Table 1). Regarding its biological activities, it is known that this peptide promotes smooth muscle contraction, reduces blood pressure in intense compensatory tachycardia, as well as assists in the release of antidiuretic hormones with consequent antidiuresis (Anastasi and Erspamer-Falconieri, 1970; Erspamer-Falconieri et al., 1970). Additionally, in rats, this peptide has been shown to increase the plasma release of corticosterone, catecholamines and glucose, as well as the release of β-endorphins (Mathison and Solomos, 1985).

#### 2.2.6 Bradykinin

Bradykinin-related peptides (BRPs) are a widespread class of amphibian skin peptides that mimic the actions of vertebrate bradykinin hormones, and are secreted in so-called granular or serous glands (Raaymakers et al., 2018). As *Phyllomedusa* species present a great richness of peptides in the tegument, it is suggested that they may increase the absorption of bradykinin by the epithelium, thus accelerating the predator intoxication process, as reported by Raaymakers et al. (2018) for *Bombina orientalis*. For *P. bicolor* phyllokinin, it is the only substance of this family to be identified from its tegument, and the use of BRPs in anti-predator defense events is unknown (König et al., 2012).

Phyllokinin has 11 amino acid residues in its primary structure, and has a sulfated tyrosine amino acid as a C-terminal residue (Anastasi et al., 1966) (Table 1). Pharmacologically, this peptide has been shown to be more potent than bradykinin in lowering blood pressure when tested in dogs; an activity that decreases considerably when the post-translational modification is not present (Anastasi et al., 1966). Regarding the kambô ritual, it is known that this peptide causes some symptoms such as increased heart rate, heat flushing, and redness of the skin (Thompson and Williams, 2022).

#### 2.2.7 Deltorphins

Deltorphins are a small family of short-chain peptides, whose members have between six and seven amino acid residues in their primary structure (Erspamer et al., 1989). In anurans, this family of peptides is restricted to the genus *Phyllomedusa* (mainly *P. bicolor* and *P. sauvageii*) (Erspamer et al., 1989). Peptides of this series are recognized for having high affinity and selectivity for δ-opioid receptors, which make them promising analgesic substances, since they have a potency that is 4,000 times greater than morphine, and 40 times greater than endogenous β-endorphin receptors (Esparmer et al., 1989). These results were corroborated by *in vivo* studies, indicating that deltorphins have high penetration rates of the blood-brain barrier (Fiori et al., 1997). The small size of deltorphins (also dermophins) may be related to their functions and/or their biological benefits in humans. Specifically, the small peptides present in *P. bicolor* are the most potent analgesic compounds; however the relationship between the size and their biological benefits remains unclear.

The first deltorphin was discovered in *P. sauvageii* (Montecucchi et al., 1981a) and served as the basis for the designation of analogous peptide sequences later described in *P. bicolor*. An interesting feature of this initial discovery was that this peptide has the second amino acid residue (methionine) with inverted stereochemistry, presenting itself in the D configuration (Table 1).

In 1989, Erspamer et al. (1989) isolated two heptapeptide analogs from the cutaneous secretion of *P. bicolor,* which were named [D-Ala2]-deltorphin I and [D-Ala2]-deltorphin II. Unlike deltorphin, they have the substitution of methionine for alanine at position 2 of the primary sequence and two valine residues in a row in the C-terminal. Another difference between these peptide analogs is the substitution of an amino acid residue at position 4 of the primary structure, in which [D-Ala2]-deltorphin I has a glutamic acid, and [D-Ala2]-deltorphin II has an aspartic acid (Erspamer et al., 1989). In the same study, they demonstrated an affinity of these peptides for opioid receptors that is 10–200 times higher than synthetic enkephalin.

#### 2.2.8 Dermophins

Dermorphins are opioid peptides, and were first isolated from *P. sauvagii* (Montecucchi et al., 1981a), *P. burneisteri*, and *P. rohdei* (Montecucchi et al., 1981b; Broccardo et al., 1981). As observed in deltorphins, they have high affinity and selectivity for opioid receptors, having been shown to produce analgesia in both humans and animals (Negri, 1992). However, the affinity and selectivity of dermorphins are for µ receptors, whereas deltorphins are specific agonists of δ receptors (Richter et al., 1990). When compared to morphine, dermorphins have a potentially more favorable adverse event profile as it is a selective agonist at the Needs correction to ϻ-opioid receptor (MOR), but does not have the relevant affinity for the ƙ-opioid receptor (KOR) (Pescatore et al., 2015; Hesselink et al., 2018b). In preclinical studies on analgesic activities with the intracerebroventricular application of the peptide dermorphin, a better performance than morphine was observed, and showed more prolonged and potent effects (Broccardo et al., 1981). Peptides of this class tend to affect the central nervous system, causing respiratory depression, in addition to high dependence on continued consumption in the kambô ritual (Nakata et al., 1986).

In *P. bicolor*, three new substances analogous to dermorphin have been described, with cDNAs cloned directly from the skin. These were named by Richter et al. (1990) as new dermorphin I, new dermorphin II, and new dermorphin III. The new analogs I and III are presented as heptapeptides, while the new analog II presented five amino acid residues in its sequence (Table 1). Unlike dermophin, they have a conserved C-terminal and, although deletions and substitutions are observed along their chain, it is possible to note that they share a similar sequence in the three amino acids of the N-terminal portion (Tyr-D-Ala-Phe).

After the chemical synthesis of these predicted peptides, they were evaluated against opioid receptors, in which only the pentapeptide showed activity, but with an IC_50_ (half maximum inhibitory concentration) that was 10 times lower than that of dermorphin (Richter et al., 1990). Subsequently, two new heptapeptides of the dermorphin class were isolated from the skin of *P. bicolor* (Mignogna et al., 1992). The first is called [Lys7]-dermorphin, which differs from the model peptide of the class by the presence of lysine at position seven (Mignogna et al., 1992). On the other hand, the peptide [Trp4,Asn7]-dermorphin has two modifications, a tryptophan amino acid residue at position 4 and an asparagine at position 7 (Table 1). In the position 2, these two analogs possess the amino acid alanine in the L configuration, as well as the conserved C-terminal (Mignogna et al., 1992). Like other peptides of this type, these peptides also showed affinity towards opioid receptors; however, the evaluation showed that amidated derivatives tend to be 2 to 4 times more potent than the versions without post-translational modification (Mignogna et al., 1992).

#### 2.2.9 Caeruleins

This family is restricted to two peptides, and only the nonapeptide phyllocaerulein (Anastasi et al., 1969) has been characterized in the species *P. bicolor*. It is an analog of the caerulein peptide, and differs in that it has one less amino acid, but maintains all the characteristics of its congener, such as composition and the two post-translational modifications: tyrosine with sulfate and the C-terminal is amidated (Anastasi et al., 1969). Another unusual aspect of this peptide is the presence of the amino acid pyroglutamic acid as an N-terminal residue. Phyllocaerulein is considered the most abundant peptide, and co-responsible (along with sauvagine) for the gastrointestinal actions observed in the kambô ritual, in particular, nausea, vomiting and the drop in blood pressure (Thompson and Williams, 2022).

#### 2.2.10 Bombesins

This class of peptides has nine amino acids, and the sequence has pyroglutamic acid as an N-terminal residue, as well as an amidated C-terminal (Table 1). Its occurrence in *P. bicolor* is limited to trace amounts, of which the phyllolitorin peptide stands out (Erspamer et al., 1985). Regarding their pharmacology, they tend to stimulate the secretion of gastric acids and increase pancreatic secretion, since they are related to the gastrin-releasing peptide. They also have been described as peptides that are capable of suppressing alcohol and food consumption after *in vivo* evaluation in chicks (Cline et al., 2010). It is worth mentioning that there are contradictions about the presence of bombesines in the secretions of *P. bicolor* (Thompson and Williams, 2022). However, research has also linked the presence of phyllolitorin with the chills observed in humans during the kambô ritual (Esakov et al., 1990).

## 3 *Kambô* ritual: From traditional use to application in large urban centers

Toads, frogs, and treefrogs are animals that are often present in folklore and culture almost everywhere and are associated with rainfall and fertility (Lima and Labate, 2007). The *kambô*, kapum, or toad vaccine (“vacina do sapo”, in Portuguese) is a purification ritual and is associated with healing rituals in the Amazon rainforest and urban centers around the world (Rudgley, 1993; Lima and Labate, 2007; Haddad and Martins, 2020). The kambô ritual is traditionally performed by shamans to purify the body, increase physical strength and sexual stamina, and ward off “panema” (bad luck and a type of weakness) (Balée, 2004; Lima, 2005; Bernarde and Santos, 2009; Labate and Lima, 2014). It is traditionally used by groups of native hunters in the southwest of the Amazon; among them, the Yawanahua, Kaxinawá, Matsés, Mayoruna, Yawanawá, and especially the Katukina (Daly et al., 1992; Hesselink, 2018a). Natives of the Pano language also apply kambô to the dogs they use during hunting (Souza et al., 2002). From the ethnological literature, there is a record of the use of kambô in more than 15 native groups, belonging to five linguistic families (Pano, Aruak, Arawa, Tikuna, and Tupi-Guarani) located in Bolivia, Brazil, Colombia, Peru, French Guiana, Suriname, and Venezuela (Lima, 2000; Lima, 2005; Lima, 2008; Lima, 2009; Lima, 2012; Lima, 2014a; b; Ribeiro, 2017).

**FIGURE 5:**
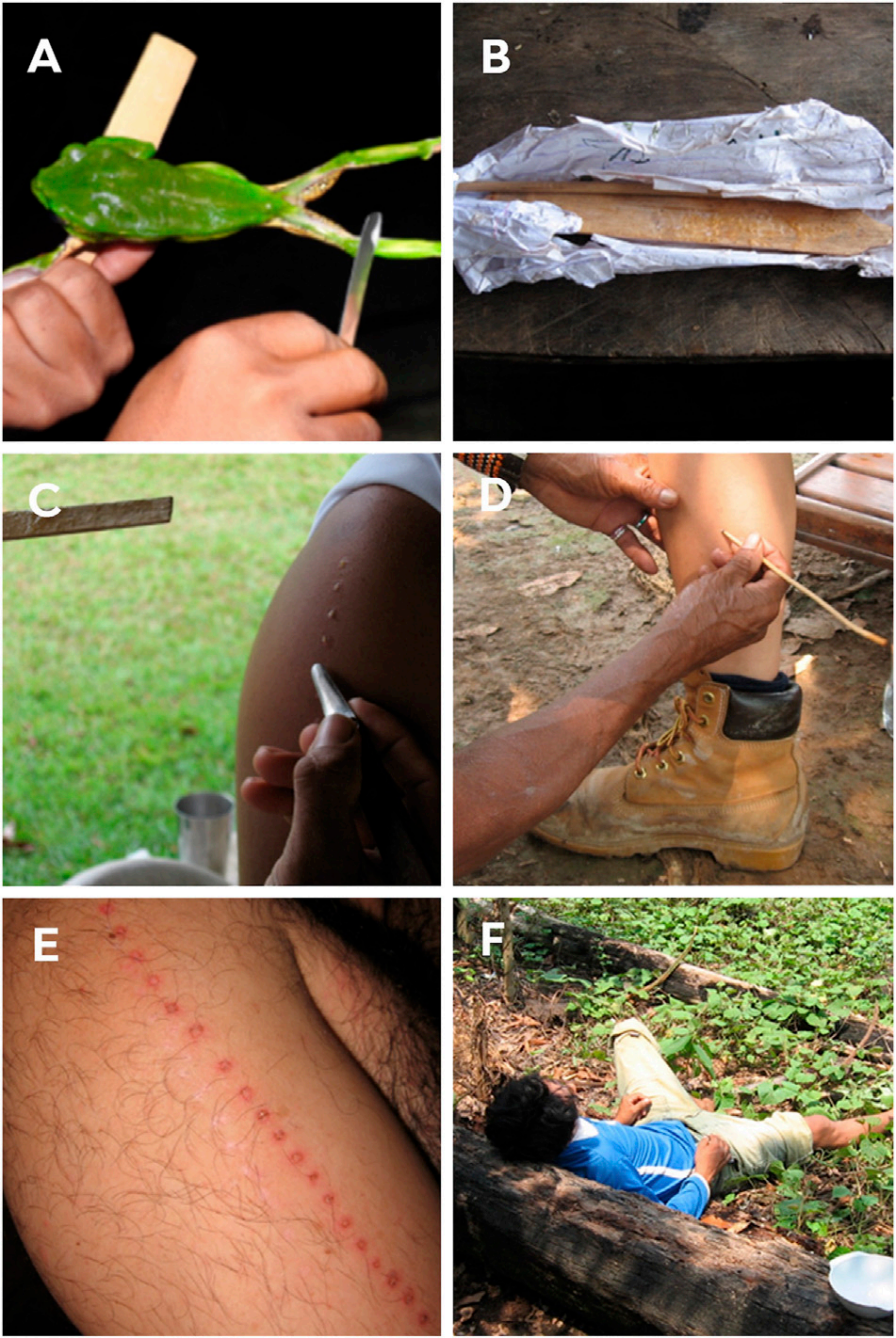
**(A)** Removal of skin secretion from the kambô frog (*P. bicolor*) by scraping the frog with a wooden rod, for later use in kambô rituals. **(B)** Skin secretion is stored for later use. **(C)** Application of secretion to the arm of a man. **(D)** Burns being made with a thin vine (“titica”) on the leg of a woman for subsequent application of the secretion. **(E)** Marks on the arms of a man right after participating in the kambô ritual. **(F)** Reaction after the application of secretion. Participants report apathy, lethargy and the urge to vomit. Photos: Paulo Sérgio Bernarde.

In this ritual, the shaman healer burns the participant and applies the *P. bicolor* secretion to the wound (Daly et al., 1992). To collect the venom, they go in search of the frog at dawn by following the characteristic sound. Due to the slow movement of the frog, it is easily captured (Daly et al., 1992; Den Brave et al., 2014). After capture, the amphibian is stretched in the shape of an “x” on crossed branches with the hind and front legs tied together (Daly et al., 1992; Den Brave et al., 2014); however, some applicators extract the poison just by handling the animal (Figure 5A). The collector teases the amphibian with pokes so that the skin secretion is released. The secretion produced is then collected by carefully scraping the frog’s skin with a wooden rod and then it is stored in straws for later use (Figures 5A,B). The frog is released after collecting the secretion and, in general, it is not customary to harm the frog or remove an excessive amount of venom or even leaving the animal trapped for a long time (Den Brave et al., 2014). Native collectors believe that hurting them can irritate animal spirits (Daly et al., 1992; Lima, 2005; Den Brave et al., 2014; Hesselink, 2018b; Majić et al., 2021).

The burns are made in the form of points with the aid of a thin vine (called “*titica*”) (Figure 5D). The number of points and the place of application are varied and are related to the body part required for activities performed by men and women; and also consider how many times the participant has been submitted to the kambô ritual (Lima, 1994; Hesselink, 2018b). Katukinas apply the secretion to the arms (deltoid) (Figures 5C,E) and chest of men. To women, it is applied to the back of the legs (calf) (Figure 5D), due to the need to strengthen the body part most used to their social role, and the participant can receive more than a hundred “points” at once (Bernarde and Santos, 2009). In the past, triple this number could be applied. Kaxinawá use the secretion in smaller amounts on the legs or arms; they apply 2 to 10 “points”, whereas the Yawanawá apply 50 to 60 “points” (Lima, 1994; Lima, 2000; Lima, 2005; Lima and Labate, 2008). A recent study carried out with non-native populations from the Western Amazon, Rondônia, Brazil, showed that women receive fewer “points”, with an average of 8.2 and 10.3 “points” in the first and second application, respectively, while men receive 8.6 and 11, respectively.

Applications of the secretion usually take place at dawn and participants are recommended to drink approximately one to 2 L of a fresh manioc drink or just water and also fast from the night before. With a full stomach, the participant usually feels the urge to vomit (Ribeiro, 2017). It is reported that the reaction is induced within minutes after the application of a dose. Reactions are often strong and include tachycardia, sweating, and severe vomiting, though generally subside in about 60 min, followed by a state of apathy and dowsiness (Figure 5F) that can last from one to several minutes, days. Subsequently, however, the participant reports greater resistance and clarity of thoughts (Daly et al., 1992; Hesselink, 2018c; Thompson et al., 2022).

Anthropological, biochemical and pharmacological studies have been carried out; however, to date, little is known about the kambô ritual (Anastasi and Erspamer-Falconieri, 1970; Carneiro, 1970; Lima and Labate, 2008; Hesselink, 2018a; Hesselink and Winkelman, 2019). The first ethnographic observations of the kambô ritual in native populations of the upper and middle Juruá were carried out by the French missionary Constantin Tastevin in 1925 (Tastevin, 1925)^.^. It is suggested that the primitive and mythological use of the secretion is related to a shaman of the Kaxinauá tribe in Brazil who, seeing many seriously ill natives and not being cured in rituals with ayahuasca, received a message to enter the forest. There he found a deity holding a green frog, and it was revealed to him how to remove the secretion from the frog and how to apply it (Hesselink, 2018c). The ritual was successfully performed and incorporated into the routine of the Kaxinauá and other tribes (Lagrou, 1991; Lima and Labate, 2008; Venâncio and Melo-Sampaio, 2010; Hesselink, 2018c). From the 19th century onwards, the coexistence between native people and the rubber tappers in the Amazon region, but specifically in the Juruá valley, resulted in a rich exchange of knowledge and practices, and, especially, the incorporation of the kambô ritual by riverine and rubber tappers (Balée, 2004). Francisco Gomes Muniz is credited with the expansion of the kambô ritual among the rubber tappers of a tributary of the Juruá River, in Acre, and the first applications of the secretion in urban centers (Lima and Labate, 2008; Hesselink and Winkelman, 2019).

During the 21st century, the practice has expanded to other neoshamanic circuits, and is currently widespread in therapy clinics, especially those linked to alternative therapies and to Brazilian ayahuasca religions, that is, among supporters of *Santo Daime* and *União do Vegetal* (Lima, 2005; Lopes, 2005; Lima and Labate, 2007; Lima and Labate, 2008; Bernarde and Santos, 2009). During its expansion to urban centers, the kambô ritual, however, was transformed into therapeutic approaches and a neoshamanic healing ritual, a process that was labeled as “shamanization of kambô”, but there is no knowledge of the ritual association between both practices (Lima and Labate, 2007; Hesselink, 2018c; Schmidt et al., 2020; Majić et al., 2021).

The expansion of the kambô ritual among non-native people in the Americas and Europe (Carneiro, 1970; Leban et al., 2016; Pogorzelska and Łapiński, 2017; Aquila et al., 2017; Li et al., 2018) has brought to the fore the discussion on issues related to traditional knowledge, in particular, the concern about the responsibility of traditional connoisseurs for the incorrect application of the secretion by non-traditional populations. Silva et al. (2019) warn that, in regions far from where the secretion is traditionally used, this vaccine could be administered by professionals who would not have the same experience as the traditional population that applies it, thus presenting health risks. Among non-native westerners, the kambô ritual is performed in a ceremonial context, different from what happens among native people, with the presence of singing, musical instruments, incense burning and prayers, tobacco snuff (containing *Nicotiana rustica*) and sananga (eye drops made from *Tabernaemontana undulata*), both traditional medicines, are often administered in the vicinity of the kambô ritual (Thompson and Williams, 2022). The sale and application of the secretion can lead to serious health risks for users. In 2004, the Brazilian National Health Surveillance Agency determined the suspension of advertising of this therapeutic alternative, since there is no scientific proof that ensures the quality, safety, and efficacy of the secretion for any type of disorder, imbalance, or treatment of any acute and chronic disorders (ANVISA, 2004).

Added to the non-traditional use, questions related to biopiracy have also been raised (Daly et al., 1992; Lima, 2014a; Den Brave et al., 2014; Pogorzelska and Łapiński, 2017; Hesselink, 2018c; Peleg Hasson et al., 2021). Dried secretions of *P. bicolor* on wooden sticks are commercially available, as “kambô sticks”, in markets and on the internet. Although the use of the secretion appears to have remained sustainable, the expansion of the use of the secretion in recent years could represent a significant environmental impact and the potential decline of the species (Cunha, 2009; Martins, 2010; Ribeiro, 2017). In 2003, the Katukinas, guided by cacique Fernando, denounced the misuse of the secretion and accused the pharmaceutical industry of having patented the kambô peptides, in addition to claiming the rights to the medicinal knowledge of the secretion. In 2004, an alliance between the Katukinas and the Brazilian government was formed to guarantee that the profits generated with the development of the secretion would benefit Brazil. This gave rise to the field project that aimed to bring together natives and researchers, mainly aiming at the regulation of its use by non-native people and the valorization and economic use of traditional knowledge regarding the use of the secretion. However, the project did not get off the ground due to the complexity of issues involving ownership and benefit-sharing (Lima, 2005; Lima, 2014b). From 2002 to 2018, 11 international patents inspired by traditional knowledge and natural genetic resources were approved, and all were issued to countries in the northern hemisphere, which may reflecting the appropriation of natural genetic resources and traditional knowledge (Shiva, 2001; Feres, 2022).

## 4 Medical aspects of *P. bicolor* envenomation

The reasons related to the use, structure, and organization of the rituals and the psychological effects of the secretion in western users are scarce and remain anecdotal (Lima and Labate, 2008; Majić et al., 2021). Majić et al. (2021) carried out an Internet-based survey investigating different aspects of use of the secretion, and observed a multitude of motivations for its use, including general healing, detoxification, and spiritual growth. According to Bernarde and Santos (2009), the reasons that lead to use by non-native populations from the Western Amazon, Rondônia, Brazil, are, in general, curiosity and health problems such as rheumatism and diabetes. Benefits to mental and physical health, including treatment of different pathologies, such as addictions, depression, chronic pain, autoimmune disorders, Hashimoto’s thyroiditis, celiac disease, post-traumatic stress disorder, diabetes, infectious diseases, cancer, hypertension, and other health conditions, have been suggested (Hesselink, 2018a; c; Silva et al., 2019; Majić et al., 2021; Thompson and Williams, 2022). Although palettes containing frog secretions can still be ordered via websites without prescriptions, benefits and important biological effects have not been demonstrated in the literature, and it has not yet been officially recognized as a drug. The beneficial effects have not been scientifically tested in randomized controlled trials, so the curative effect may just be a placebo effect (Aquila et al., 2017).

The mechanisms of action and the effects of the secretions from *P. bicolor* are not completely understood, especially due to the diversity of toxins present in the secretion. *In vitro* cytotoxic and antitumor effects on different cells have been identified, as well as antibacterial and antiprotozoal properties, besides substances with high affinity for µ-opioid receptors (Negri et al., 1992; Erspamer et al., 1993; Leite et al., 2005; Hesselink and Schatman, 2018) and dilation and increased permeability of vessels and the blood-brain barrier (Erspamer et al., 1989; Erspamer et al., 1993; Daly et al., 2009; Van Zoggel et al., 2012; Li et al., 2018). Overall, frog secretions tend to cause severe adverse side effects, including death. Short-term effects appear within minutes and include gastrointestinal problems, characterized primarily by severe vomiting and diarrhea, urination, sweating, and tachycardia (Erspamer et al., 1989; Erspamer et al., 1993). Although, some effects are considered mandatory symptoms because it is related to cleansing the body of bad influences or toxins (Hesselink, 2018d), these effects are generally dose-dependent (Daly et al., 1992; Li et al., 2018). Increased physical strength, stamina, and the ability to cope with stressful situations are prime examples of the secretion’s late effects. Other delayed effects are hallucination, drowsiness, dizziness, euphoria, and sedation (Erspamer et al., 1989; Erspamer et al., 1993). These effects are mediated by the actions of peptides present in the skin secretion such as phyllokinin, adrenoregulin, sauvagine, phyllocerulin, phyllomedusin, dermorphins, and deltorphins (Li et al., 2018; Byard, 2019). Some researchers have attempted to understand the full effect of the crude secretion, but with few and uncertain results (Thompson et al., 2022; Thompson and Williams, 2022). Generally, most studies focus on the secretion-derived peptides, with individual effects of the main isolated components (Table 2).

**TABLE 2:** Effects and recommendations reported in the literature related to the secretion from *Phyllomedusa bicolor*.

age, sex	country	recommendation	signs and symptoms	symptoms of intoxication	outcome	reference
41 years, woman	Chile	Depression	Unresponsive, with extreme hypotonia of the limbs and hypoventilation	Severe neurologic effects, rhabdomyolysis and renal failure (on the second day)	Discharge	Alamos et al. (2020)
46 years, man	Bolivia	-	Subcutaneous lipoma	-	Discharge	Den Brave et al. (2014)
42 years, man	Greece	No recommendation	-	Left ventricular hypertrophy	Death	Aquila et al. (2017)
44 years, woman	Slovenia	-	Nausea and vomiting, confusion, lethargy, muscle weakness, spasms and cramps, seizure, decreased consciousness level and short-term memory loss	Neurological symptoms and hyponatremia result of inappropriate antidiuretic hormone secretion	Discharge	Leban et al. (2016)
34 years, mean	-	Abstinence from drinking, smoking, and to purify the body	Icterus, skin itching, weakness, and pain in the upper abdomen	Toxic hepatitis	Discharge	Pogorzelska and Łapiński (2017)
32 years, woman	-	-	Nausea, vomiting, and abdominal discomfort	Hyponatremia	Discharge	Kumachev et al. (2018)
24 years, woman		-	Nausea, vomiting, flushing, facial swelling, altered mental status, and extreme agitation	Prolonged toxicity (maintenance of symptoms after 22 h of use)	Discharge	Li et al. (2018)
33 years, woman		Alleviate chronic pain	Paranoia, anxiety, bizarre delusions, labile mood, and panic attacks	Psychosis	Discharge	Roy et al. (2018)
58 years, woman	-	Cholangiocarcinoma	Tachycardia, tachypnea, impaired liver cholestatic enzymes, and enlargement of lymphadenopathy mimicking disease progression	Systemic inflammatory response syndrome mimicking disease	Death	Peleg Hasson et al. (2021)
33 years, woman	Brazil	No recommendation	3-week history of asthenia, malaise, myalgia, and proximal muscle weakness, particularly in the lower limbs	Dermatomyositis	Discharge	De la Vega et al. (2020)
41 years, woman	Chile	-	Vomiting, profuse diarrhea, quantitatively compromised consciousness, and tonic-clonic convulsions	Hyponatremia	Discharge	Campodónico et al. (2019)
woman	-	-	Vomiting and liquid stools that were self-limited	Hyponatremia result of inappropriate antidiuretic hormone secretion	Discharge	Agüero-González et al. (2019)
62 years, woman	-	-	Shortness of breath, epigastric abdominal pain, nausea, and non-bloody emesis	Led to pneumothorax, septic shock, and esophageal rupture	-	Gonzaga et al. (2020)
37 years,woman^a^		Depression	Visual hallucination, agitation, tremors of extremities, oral paresthesia, seizures, nausea, vomiting, sweating and five skin lesions on the leg	Neurological symptoms	Discharge	Morais et al. (2018)

a Concomitant ayahuasca and *P. bicolor* secretion use.

The most common causes of illness and death after kambô rituals are associated with the depressive impact of opioid derivatives on the CNS and the effects of toxins on the cardiovascular system. Kidneys, pancreas and liver can be damaged (Hemingway et al., 2009; Pogorzelska and Łapiński, 2017). There are a few reports of intoxication with both *P. bicolor* secretion and ayahuasca that led to hospitalization (Morais et al., 2018; Campodónico et al., 2019). However, there are some reports of hospitalization due to intoxication with just the secretion that presented severe side effects (Leban et al., 2016; Hesselink, 2018d; Campodónico et al., 2019; De la Vega et al., 2020; Sacco et al., 2022). In a study with 127 participants who used the secretion, Thompson et al. (2022) observed non-severe physical responses, such as facial swelling, diaphoresis, bowel movements, and syncope, and increasing the dose was responsible for the increase in facial swelling and sweating. Although other studies have shown that the kambô ritual can increase the risks of damage from hyponatremia, asphyxia from vomiting, and injury from syncope and death (Menocchi, 2008; Labate and Lima, 2007; Aquila et al., 2017; Sacco et al., 2022), due to the high concentration of bioactive peptides and potency to certain receptors, the effects can be misinterpreted as intoxication or a massive allergic reaction (Hesselink, 2018d).

## 5 Study perspectives

As a rich source of peptides, *P. bicolor* continues to entice researchers in the medical and biotechnological fields due to its vast chemodiversity (as yet not fully known), as well as for all its pharmacological potentials. Regarding this potential, there is a need for studies that aim to further elucidate the mechanisms of action of some of the peptides, especially the opioids that have the potential to be applied in the treatment of chronic diseases. In parallel, the results of studies that indicate antibiotic, antineoplastic, antiviral, immunomodulatory and antiparasitic potential should be further investigated. This need for further studies is supported by the inherent characteristics of these peptides, such as low molecular weights, high activities, low cytotoxicity, high water solubility and rapid absorption. In this way, it is expected that, in the future, the chemistry of secretions from *P. bicolor* will advance towards useful and safe treatments, where once again traditional knowledge helps future generations.

## 6 Final remarks

The species *P. bicolor* is prevalent in the Amazonian biome, where it is used by the indigenous populations in the folk and popular sectors of healthcare. Currently, its use has spread to other populations, still with the original objectives of prevention and treatment of a series of illnesses and traditional syndromes, as well as for use in certain religious cults. The expansion of its use has raised concerns about biopiracy, with reports of use in European countries and the United States. Excessive use or use by persons with prior health problems can result in critically-ill envenomation and deaths. There are no management protocols for these cases of envenomations, and supportive care has been adopted in the few reports found in the literature. The richness of the secretion’s bioactive peptides with different biological activities is responsible both for the signs and symptoms of their popular use and for the envenomations. Several studies have been carried out to better understand the chemical nature of these peptides and their biological effects from *in vitro* and limited *in vivo* data. From a biotechnological perspective, the antibiotic, antineoplastic, antiviral, immunomodulatory and antiparasitic potential activities are of interest. (Bahar and Ren, 2013, Mor et al., 1994c, Vanhoye et al., 2004).
